# People are less likely to selfishly deceive those who achieved status through virtue rather than dominance or competence

**DOI:** 10.1098/rsos.250552

**Published:** 2025-11-26

**Authors:** Sarah Boukarras, Valerio Placidi, Michael Schepisi, Vanessa Era, Maria Serena Panasiti, Matteo Candidi

**Affiliations:** ^1^ Department of Psychology, Sapienza University of Rome, Italy; ^2^ IRCCS Santa Lucia Foundation, Rome, Italy; ^3^ Centre for Neuroscience, University of Camerino, Italy

**Keywords:** social status, moral behaviour, deception

## Abstract

Moral behaviour varies across contexts, yet the influence of the recipient’s social status—the person towards whom the behaviour is directed—remains largely underexplored. The strategies used to achieve status can vary substantially and play a crucial role in shaping social perception and behaviour. For instance, dominance-based status triggers negative evaluations, whereas people who attain status through competence or virtue often gain respect and admiration. This preregistered study (*n* = 151) investigated how an opponent’s social status (high, middle or low) and the strategies used to achieve it (dominance, competence or virtue) influenced participants’ tendency to lie for self-gain during a card game. Results indicate that participants were significantly less likely to lie for self-gain to virtuous high-status opponents compared to dominant or competent ones. Dominance-based high-status opponents elicited negative emotions (e.g. anger, disgust), while virtuous and competent opponents inspired admiration and respect. These findings highlight that moral behaviour is shaped by both the status of the recipient and the means by which it was acquired, with honesty more likely directed towards virtuous high-status individuals. This research has implications for education and leadership, suggesting that strategies involving virtuous and prosocial behaviours can protect leaders from being deceived by their followership.

## 1. Introduction

Lie-telling, the deliberate act of manipulating or concealing information from others [1], is a pervasive aspect of human behaviour, despite being generally disapproved. Among the various forms of deception, self-serving lies—those that benefit the liar at the expense of the recipient—are the most condemned and pose the highest risk of reputational damage [2]. Despite this, such behaviour is remarkably common and can be found across a wide range of social contexts, from the workplace to intimate relationships. For instance, consider Jane, who applies for a high-level job requiring proficiency in a specific software. To secure the position, she falsely claims expertise on her resume and during the interview. Similarly, John, who is in a long-term relationship with Louise, begins seeing another woman and deceives Louise about his whereabouts. Both Jane and John face a moral conflict: lying to obtain or maintain a reward (a prestigious job or a double relationship) versus adhering to ethical principles and social norms [3]. How individuals resolve this conflict, or, in other words, why some people lie and others do not, probably depends on multiple factors. Previous research has highlighted the role of both contextual (e.g. reputation risk) [4] and individual-level predictors of deception, such as personality traits [5,6], mindfulness levels [7] and bodily awareness [8].

Conversely, surprisingly little attention has been paid to the characteristics of the potential recipient of the lie—in the examples above, the hiring manager and Louise. At a theoretical level, the moral exclusion account [9] proposes that individuals adjust their moral compass depending on who the recipient of the morally relevant act is. The theory suggests that when people see others as unworthy of fairness, empathy or justice, they become more likely to tolerate or even support unethical actions against them. It is however unclear whether this distinction translates from moral judgements to first-person behaviours and, in particular, to deception. Evidence from experimental research reveals a fragmented picture: while some studies found no difference in the number of lies told to ingroup versus outgroup opponents (e.g. [10]), others observed an increase in self-serving lies to strangers, compared to close friends [11,12], and to cold (compared to warm) opponents [13].

One interesting question is whether people adjust their moral behaviour depending on the social status of the recipient. Indeed, social status, defined as the amount of consideration and respect that a person receives from others [14,15], is a powerful determinant of interpersonal behaviour [16] and may shape moral behaviour in opposing ways. High status individuals may elicit greater deference, respect or fear of reputational consequences, thus discouraging dishonest behaviour. Given their higher instrumental value [17], they might be more likely to be included within the ‘scope of justice’ than low-status individuals [9]. Conversely, low-status individuals might be perceived as less socially relevant, making them more vulnerable to deception. Despite the growing interest in the interplay between social status and morality [18–20], whether the status of the recipient influences lying behaviour has received little attention. One exception is a recent study reporting an increase in self-gain lies to opponents depicted as having a high- (compared to low-) status job in a company [21]. However, since the lies in that study led to a monetary loss for the opponent, it is possible that participants simply refrained from taking money away from the less wealthy opponent. The present study builds on [21] to explore how other status dimensions—beyond wealth—may influence lying behaviour. In fact, although socioeconomic level is an important aspect of social identity, status-based hierarchies extend far beyond mere economic distinctions.

Previous research indicates that high-status individuals catalyse attention [22–24], are positively evaluated [25,26] and benefit from preferential access to resources [27], mating opportunities [28,29] and from better health and longevity [30]. However, while status is often viewed as a universally positive and highly desirable end-state, the strategies individuals use to achieve it can vary considerably [16], influencing how status holders are perceived within social groups. Leading status theories propose either a two-way (dominance-competence) or a three-way model (dominance, competence and virtue) of status achievement. Dominance-based tactics entail the use of force, intimidation and threat to gain influence in a group [15,25,31], while competence-based status is voluntarily afforded by others to individuals who demonstrate outstanding abilities in a socially valuable domain [32–34]. Finally, the display of virtuous behaviour beyond mere conformity to norms can also lead to high status [35–40]. Although dominance, competence and virtue are all equally valid strategies to achieve a high standing within a group, there is evidence that, while competent and virtuous high-status individuals are positively evaluated and liked more than lower-status ones [15,38,41–43], dominant high-status individuals are substantially disliked by others [15]. Furthermore, while dominance-based status is attained by instilling fear, competence and virtue supposedly lead to status through respect and admiration, respectively [40]. Building on this framework, we hypothesize that lying behaviour will be differentially influenced not only by the recipient’s status level but also by the status-seeking strategy they have adopted. Specifically, we hypothesize that individuals who attained high status within a group through prosocial acts (virtue) or by demonstrating superior knowledge (competence) would be less likely to be targeted by immoral behaviours, compared to those who achieved their status through dominance, a strategy that may be perceived as threatening and elicit negative feelings.

In the present study, participants were engaged in the ‘temptation to lie’ card game (TLCG; [2]), an ecologically validated task in which they were free to lie or tell the truth about the outcome of a card game to an opponent. They were led to believe they were playing against a group of opponents who were described as having achieved a high, middle, or low status (i.e. status level) in a previous encounter using either dominance, competence, or virtue (i.e. status dimension), see table 1 and the electronic supplementary material, table S1. We further explored the emotions elicited by the characters within each combination of status level and dimension, the effects of elicited emotion and individual differences on lying behaviour, and how reaction times (RTs) were modulated by participants’ response (lie/truth), trial outcome, status level and dimension. These exploratory analyses were aimed at providing information about the emotional and cognitive processes underlying participants’ decisions.

**Table 1.** Brief description of opponents’ behaviour in the alleged ‘previous study’*.* (Note: a full description of the opponents’ behaviour, as presented to the participants, can be found in the electronic supplementary material, table S1.)

status dimension	behaviour	frequency of behaviour		
		status level		
		high	middle	low
dominance	took the floor, showed confidence, criticized others, tried to direct the group work and impose their will. Other members were afraid of them. Was listened to and had influence on collective decisions	always	sometimes	never
competence	demonstrated superior understanding of the topics, was able to handle difficult situations. Worked quickly and effectively. Proposed appropriate solutions. Was listened to and had influence on collective decisions	always	sometimes	never
virtue	helped and assisted others sacrificing their time. Collaborated easily with others. Worked longer hours and took heavier tasks. Attempted to solve conflicts. Was listened to and had influence on collective decisions	always	sometimes	never

## 2. Methods

### 2.1. Preliminary vignette validation and pilot experiment

**Figure 1.**
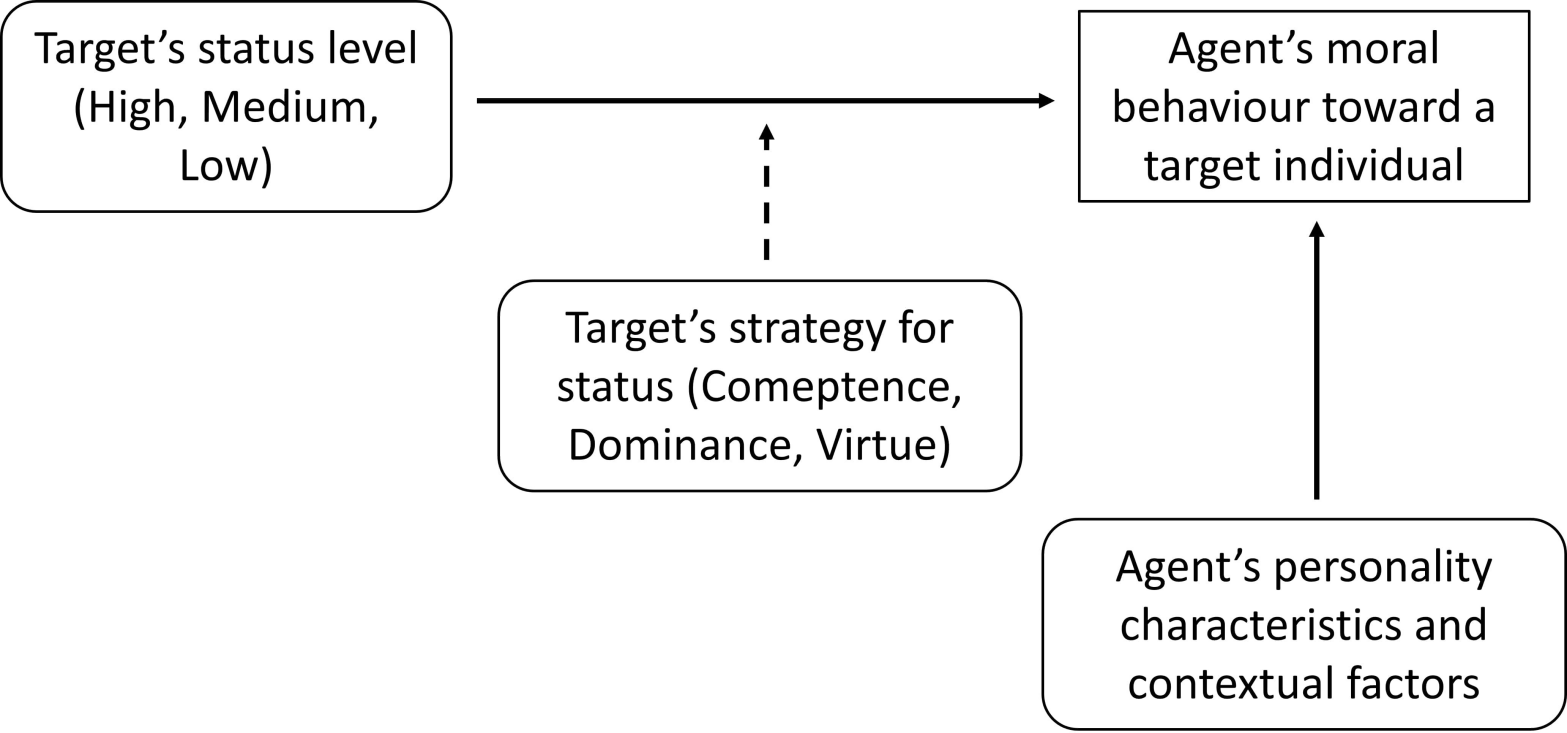
Schematic representation of the study’s rationale and objectives. Previous research has highlighted how the moral behaviour of an agent towards a target individual is shaped by contextual factors, the agent’s personality and the target’s status level (solid lines). In the present study, it is posited that the strategy used by the target to attain social status affects the relationship between their status level and the agent’s moral behaviour towards them (dashed line).

Before performing the present experiment, we conducted a preliminary validation (*n* = 68) of the vignettes describing the opponents’ profiles (see the electronic supplementary material), and a pilot experiment (*n* = 48) with the full procedure used in the present experiment (see the electronic supplementary material). From the results of the pilot experiment, we generated a set of hypotheses that were pre-registered on the Open Science Framework (OSF) platform (https://doi.org/10.17605/OSF.IO/XJKFP) and are summarized in figure 1 and extensively reported in table 2. These hypotheses were tested in §2.2.

**Table 2.** Set of hypotheses preregistered on the OSF following the pilot study. (Note: the first column table reports the hypothesis, the second specifies its predictions,and the third indicates whether the predictions were supported in the main experiment.)

hypothesis	expected differences	supported/ not
H1—directional. The predicted probability of egoistic lies to the high-status opponent in the dominance group will be higher compared to the predicted probability of egoistic lies to the high-status opponents in the virtue and competence groups and compared to the predicted probability of egoistic lies towards the low- and middle-status opponents in the dominance group	high-status_dominance > high-status_competence	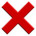
	high-status_dominance > high-status_virtue	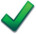
	high-status_dominance > low-status_dominance	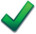
	high-status_dominance > middle-status_dominance	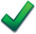
	middle-status_dominance > low-status_dominance	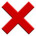
H2—directional. The predicted probability of egoistic lies towards the high-status opponent in the competence condition will be lower compared to the predicted probability of egoistic lies towards the low- and middle-status opponent in the same condition	high-status_competence < low-status_competence	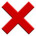
	high-status_competence < middle-status_competence	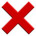
	middle-status_competence < low-status_competence	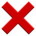
H3—directional. The predicted probability of egoistic lies towards the high-status opponent in the virtue condition will be lower compared to the predicted probability of egoistic lies towards the low- and middle-status opponent in the same condition	high-status_virtue < low-status_virtue	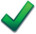
	high-status_virtue < middle-status_virtue	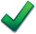
	middle-status_virtue < low-status_virtue	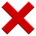
H4—directional. The high-status profile will be rated as having higher status and having received more attention compared to the low- and middle-status profiles, irrespective of the status pathway—i.e. the three pathways are equally effective for gaining status	high-status > middle-status > low-status	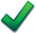

### 2.2. Main experiment

#### 2.2.1. Participants

One hundred and eighty-seven participants were enrolled in the present experiment. From this initial sample, 36 participants were excluded either because they failed the funnel debriefing (see §2.2.2), or because of technical issues (e.g. connection issues). Therefore, the final sample consisted of 151 individuals (82 females (F), two non-binary) with a mean age of 25.24 (s.d. = 3.89). This sample size (i.e. *n* = 151) was deemed adequate to detect a moderate effect size of *ηp^2^* = 0.06, with a power of 0.95 and an alpha level (two-sides) of 0.05 for a 2 × 3 × 3 design as computed by an *a priori* power analysis run on the software MorePower [44]. Participants were recruited from a laboratory database and through posts on social media, and randomly assigned to the competence (*n* = 50 (28 F), age = 25 (s.d. = 4) years), dominance (*n* = 51 (27 F), age = 25 (s.d. = 3.9) years, or virtue (*n* = 50 (27 F), age = 26 (s.d. = 3.7) years) manipulation. No differences between the groups were present in terms of age (*F* = 1.03, *p* > 0.05) and gender (*χ^2^* = 1.04, *p* > 0.05). Before taking part in the experiment, the volunteers read and signed the informed consent and were made aware that only one among the participants involved in their session (i.e. the actual participant plus six fictitious opponents), randomly chosen, would have received monetary compensation.

The experimental protocol was approved by the ethics committee of the Psychology Department of Sapienza University of Rome. The experiment was conducted online between December 2021 and May 2023.

#### 2.2.2. Procedure

**Figure 2.**
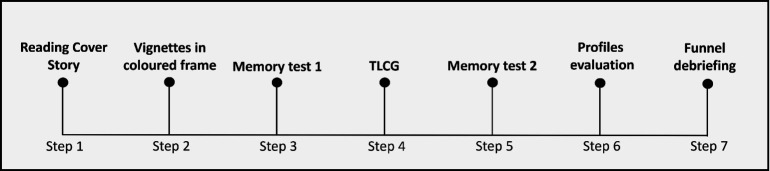
Timeline of the experimental procedure.

A schematic depiction of the experiment timeline is provided in figure 2. After having agreed to take part in the experiment, participants were sent a URL link redirecting them to a Qualtrics (Qualtrics Research Suite ©, 2020) survey where they could read the experiment description (step 1: reading cover story). As a cover story, participants were informed that the experiment intended to study the effect of social interactions on memory (see the electronic supplementary material for a full description). More specifically, they were told that they would play a card game with some individuals (hereafter, ‘opponents’) who participated in a previous study. During this alleged previous study, the opponents were involved in group work, after which they were asked to evaluate the behaviour of their peers. According to the cover story, these evaluations had been summarized in short paragraphs (hereafter, ‘vignettes’) describing the behaviour of each opponent during the alleged group work in terms of their attitude towards the other group members and their ability to handle the group work. Participants were asked to read three vignettes (step 2: vignettes in coloured frames), describing the behaviour of the high-, middle- and low-status opponents, as confirmed by both participants of the validation study (*n* = 68) and of the current one (see §2.2.3). The three vignettes were framed with different colours (i.e. sky blue, light purple and blue). Participants were asked to learn the association between vignettes and colours. For colour-blind participants, an alternative version of the frames with easily distinguishable colours (i.e. yellow, red, blue) was provided. Participants’ memory for the colour-vignette association was tested (step 3: memory test 1) to ensure that they were able to correctly identify the opponents’ identity from the frame’s colour in the main task, which is the TLCG [2]. Following instructions, participants performed the task (step 4: TLCG), which was delivered through the Psytoolkit online software (version 3.4.0, [45]), then their memory was tested again (step 5: memory test 2) to verify that they had retained the colour-vignette association throughout the task. After, participants were asked to read the vignettes again and to evaluate the opponents along multiple dimensions (step 6: profiles evaluation). Finally, three questions (step 7: funnel debriefing) were asked in the following order: (i) ‘during the game, did you have the feeling that the people you were playing with were not real?’; (ii) ‘during the game, do you think you behaved as you would have in real life?’; and (iii) ‘how much do you think your behaviour during the game reflects your behaviour in real life?’. The first two questions had a binary ‘yes/no’ response option, while for the third one participants answered using a Visual Analogue Scale (VAS) ranging from 1 (not at all) to 100 (totally). Importantly, those who answered ‘no’ to the second question (the one concerning real-life behaviour) were excluded from statistical analyses. At the end of the experiment, the hypotheses, design and real compensation probability were fully disclosed to them through a standardized debrief message (see the electronic supplementary material). Experimental sessions lasted about 50 min.

#### 2.2.3. Status vignettes

The nine status vignettes (see the electronic supplementary material, table S1), reporting the alleged behaviour of the opponents during the group work (see §2.2.2), described individuals (opponents) who obtained a certain status in the group (low, middle or high) through a specific pathway (competence, dominance or virtue). Status level was operationally defined as the amount of attention the opponent received from their peers [15] and the influence they exerted on the group. Thus, high-status opponents were described as being ‘always’ listened to by the others and having influenced ‘all’ the collective decisions (as opposed to ‘sometimes’ for the middle status and ‘never’ for the low status). The status dimension or pathway was operationalized based on the prestige-dominance account [15,33] and on the virtue theory of status [40]. The competent high-status character was presented as someone who had a ‘higher’ knowledge of the topics, solved ‘all’ the problems, ‘never’ made mistakes, was fast and efficient, and ‘always’ proposed the right solutions. Using a procedure similar to the one adopted by [40], middle and low-status characters were constructed by varying the adverbs reflecting the frequency of the described behaviours (i.e. had an ‘average’/‘lower’ knowledge, solved ‘some’/‘no’ problems, ‘sometimes’/‘always’ made mistakes and proposed the right solutions). The dominant high-status character was presented as someone who ‘always’ took the floor, interrupted other people talking, showed extreme self-confidence, was impatient, and tried to impose their will. The virtuous high-status ‘always’ worked harder than the others, helped those in need, was cooperative, and tried to restore harmony in case of disagreements. The middle and low-status characters for the dominance and virtue pathways were constructed as described for the competence one. Each participant was only exposed to profiles related to a single pathway to status acquisition, which differed in their status level (e.g. participants in the dominance group were presented with the low-dominance, middle-dominance and high-dominance profiles). Thus, status dimension (or pathway to status) varied between subjects, while status level varied within subjects.

#### 2.2.4. Temptation to lie card game

**Figure 3.**
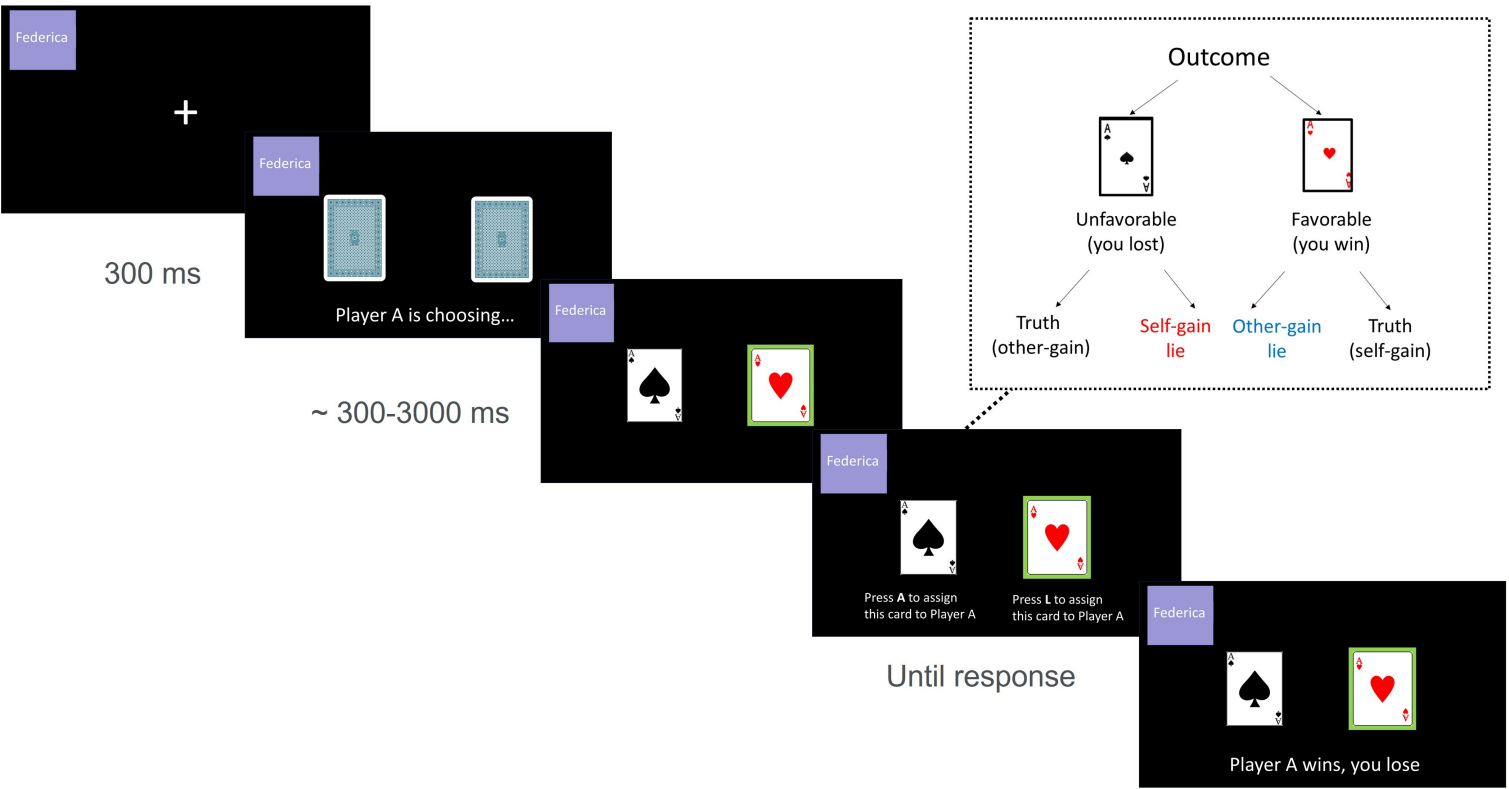
Timeline of the TLCG.

The TLCG [2] is an ecological task in which participants can decide whether to lie or tell the truth to an opponent to obtain a monetary reward for themselves or to donate it to the opponent. The opponent, whose behaviour, unbeknown to the participant, is randomly generated by the computer, is always the first to choose between two covered cards, which are presented on the computer screen (see figure 3). Importantly, participants are informed that the opponent (player A) is prevented from seeing the outcome of their own choice, which can be either the ace of hearts (winning card) or the ace of spades (losing card), and that they have to communicate the outcome to the opponent. From the participants’ point of view, ‘favourable’ trials are the ones in which the opponent selects the losing card (and, thus, the participant gets the reward), while ‘unfavourable’ trials are the ones in which the opponent selects the winning card. In both cases, the participant can either accept the outcome or, by lying, reverse it, thus winning when he/she has actually lost (self-gain lie) or losing when he/she has actually won (other-gain lie). In the present experiment, participants were told that they were playing with six fictitious opponents (three females and three males). In each trial, the name of the opponent was shown within a coloured square frame reflecting the three colours previously associated with the status vignettes. In this way, participants were able to identify the status level of the opponents just by looking at the frame (see figure 3). For each status level, one male and one female opponent were included in the TLCG. The game comprised 48 trials (eight for each opponent). Participants were informed that during each trial an indefinite amount of points would be awarded to the winner and that at the end of the game, only one among all the players would have been randomly picked to get a monetary compensation proportional to the number of points accumulated during the game. A detailed description of the task is presented in the electronic supplementary material.

#### 2.2.5. Profiles evaluation

After the TLCG, participants were asked to read the vignettes again and to rate on a 7-step Likert response scale, with 1 = ‘not at all’ and 7 = ‘a lot’, the following statements: ‘this person had a high status in the group’ (STATUS); ‘this person received attention by the others’ (ATTENTION); how much [status-enhancing characteristic] do you think this person possesses? (COMPETENCE, DOMINANCE, VIRTUE); ‘how much [emotion] does this person elicit in you?’ (ADMIRATION, ANGER, DISGUST, ENVY, FEAR, PITY and RESPECT). Participants were provided with a verbal description of each emotion (see the electronic supplementary material, table S2).

#### 2.2.6. Personality questionnaires

Participants were asked to fill in validated questionnaires in their Italian versions. The 14-item social dominance orientation (SDO; [46]) measures people’s tendency to endorse the existence of social hierarchies and to desire that the in-group prevails over out-groups. Facets of moral identity (i.e. the extent to which morality plays a central role in self-definition and behavioural regulation [47]) were measured with two scales: the moral identity questionnaire (MIQ; [48]) and the self-importance of moral identity (SIMI; [49]). Although both scales measure the level to which morality shapes self-identity, a major difference between the two is that MIQ, but not SIMI, includes a subscale that evaluates the consistency between one’s own moral beliefs and actions. Finally, participants filled in the MacArthur scale of subjective social status (SSS; [50]), which assesses a person’s perceived rank relative to others in their social group.

#### 2.2.7. Experimental design and statistical analyses

The experiment had a 2 × 3 × 3 mixed design with outcome (favourable versus unfavourable) and status level (high versus medium versus low) as the within-subjects factors, and status dimension (dominance versus competence versus virtue) as the between-subjects factor. For the TLCG, where we had the binomial dependent variable ‘lie’ (0 = truth, 1 = lie), we used a generalized linear mixed model (GLMM), which included as fixed factors outcome, status level and status dimension, while the random structure included a by-participants intercept and the slopes of outcome and status level. The characters evaluation Likert data were analysed using the align rank transform method [51] implemented through the R package ARTool [52].

## 3. Results

### 3.1. Confirmatory analyses

#### 3.1.1. Profiles ratings: attribution of status and attention

The ART ANOVAs for both attention and status ratings revealed a significant effect of status level (attention: *F*_2,294_ = 749.39, *p* < 0.0001; status: *F*_2,294_ = 822.10, *p* < 0.0001) and no significant effect of status dimension. Within-group post-hoc comparisons revealed the predicted significant high > middle > low pattern for both variables (all *ps* < 0.0001), see the electronic supplementary material, tables S3 and figure S1. Although both models revealed a significant status level × status dimension interaction (attention: *F*_2,294_ = 6.05, *p* < 0.001; status: *F*_2,294_ = 6.31, *p* < 0.0001), the only significant between-group difference was observed for the variable status between the dominant middle status and the virtuous middle status (estimate = 43.81, s.e. = 11.64, *p* < 0.05). Conversely, no differences in status and attention ratings were observed among the high-status profiles (all *ps* > 0.48) and the low status ones (all *ps* > 0.79), see the electronic supplementary material, tables S4 and figure 2. Thus, as predicted by H4, in each group, the high-status profiles received higher ratings of status and attention compared to their low and middle-status counterparts, indicating that the three pathways are equally effective for gaining status.

#### 3.1.2. Social status level and dimension impact the probability of lying

**Figure 4.**
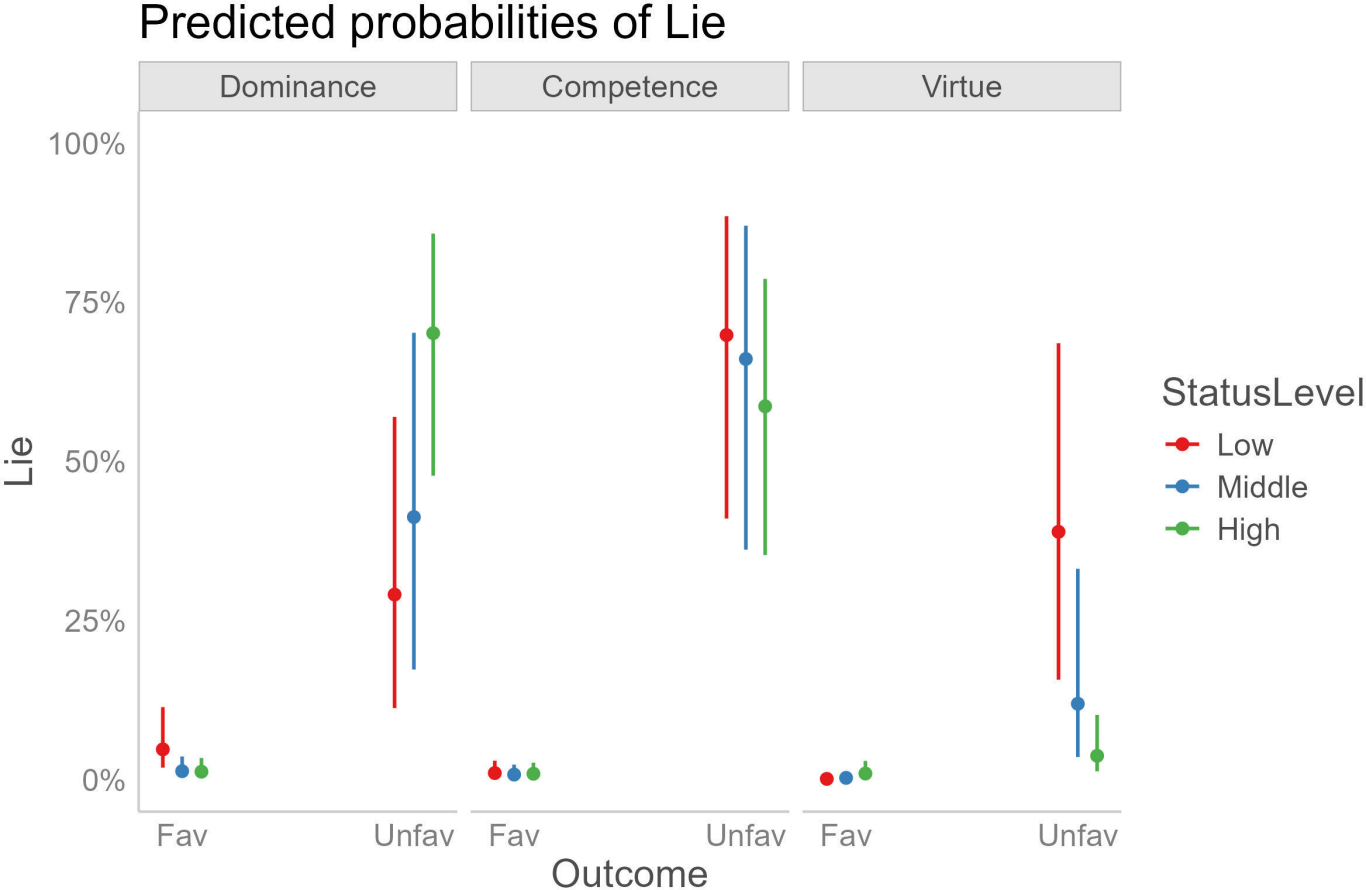
Predicted probabilities of lie in the experimental groups (dominance, competence and virtue) depending on status level and outcome. Fav, favourable outcome (win trials); Unfav, unfavourable outcome (lose trials). Lies in the favourable condition are considered to be ‘other-gain’, while lies in the unfavourable condition are ‘self-gain’.

The GLMM (conditional *R^2^*: 0.83, marginal *R^2^*: 0.26) yielded significant main effects of outcome (*χ²* = 64.29, *p* < 0.0001), status dimension (*χ²* = 14.66, *p* < 0.001) and outcome × status level × status dimension interaction (*χ²* = 37.24, *p* < 0.0001), see figure 4. Tukey-adjusted post hoc tests on the three-way interaction revealed that, as predicted by H1, the probability of self-gain (but not other-gain) lies was higher for the high-status dominant than for the high-status virtuous opponent (estimate = 4.09, s.e. = 0.78, *z*-ratio = 5.19, *p* < 0.0001). Instead, contrary to H1 predictions, no significant difference was observed between the high-status dominant and the high-status competent (estimate = −0.50, s.e. = 0.70, *z*-ratio = −0.72, *p* = .99). The probability of self-gain lies was also significantly higher for the high-status competent compared to the high-status virtuous (estimate = 3.588, s.e. = 0.79, *z*-ratio = 4.52, *p* < 0.001).

When looking at the effects of status level within each experimental group, we observed that, in accordance with H1, participants in the dominance group lied less for self-gain to the low than to the high (estimate = −1.74, s.e. = 0.48, *z*-ratio = −3.62, *p* < 0.01) and middle status (estimate = −1.20, s.e. = 0.32, *z*-ratio = −3.75 , *p* < .01) opponents, while, contrary to our predictions, there was no significant difference between the high and middle status opponents (estimate = −0.53, s.e. = 0.36, *z*-ratio = −1.46, *p* = 0.87). In the virtue group, we observed the predicted (see H3) differences in self-gain lies between the high and low status (estimate = 2.78, s.e. = 0.61, *z*-ratio = 4.55, *p* < 0.001) and the middle and low status opponents (estimate = 1.54, s.e. = 0.46, *z*-ratio = 3.31, *p* < 0.05), while the high-middle comparison was non-significant (estimate = 1.24, s.e. = 0.48, *z*-ratio = 2.57, *p* = 0.19). Contrary to our hypothesis H2, no significant status level effect was observed in the competence group (all *ps* > 0.98). Furthermore, as can be observed in figure 4, for the dominance and virtue groups, there was a reversed scale effect of status level, such that status increasingly enhanced the probability of being deceived for the dominance manipulation, while decreasing it for the virtue one. An exploratory analysis of the probability of other-gain lies revealed a significant difference between the low status dominant and the low status virtuous opponents (estimate = 3.53, s.e. = 0.88, *z*-ratio = 4.02, *p* < 0.01), indicating that participants lied more to benefit the former compared to the latter.

### 3.2. Exploratory analyses

#### 3.2.1. Reaction times model for the temptation to lie card game

We examined whether RTs (in seconds) were influenced by the experimental variables. We built an Linear Mixed Model (LMM) with response (truth or lie), outcome, status level, status dimension, their interaction as fixed effects and a by-participants random intercept. The model yielded a significant effect of outcome (*χ²* = 13.17, *p* < 0.001), indicating that participant responses were slower during unfavourable than favourable trials. Moreover, a response × outcome interaction (*χ²* = 15.33, *p* < 0.0001) showed that, when the game outcome was favourable, participants took longer to lie than to say the truth (estimate = 1130, s.e. = 299, *z*-ratio = 3.85, *p* < 0.001). Conversely, when the outcome was unfavourable, there was no difference between lies and truth (estimate = -165, s.e. = 169, *z*-ratio = −0.97, *p* = 0.763). All other effects were nonsignificant. Since RTs can be indicative of conflict processing, our results suggest that participants experienced more conflict before lying to benefit the other than before lying for their personal gain.

#### 3.2.2. Profile ratings of elicited emotions

**Figure 5.**
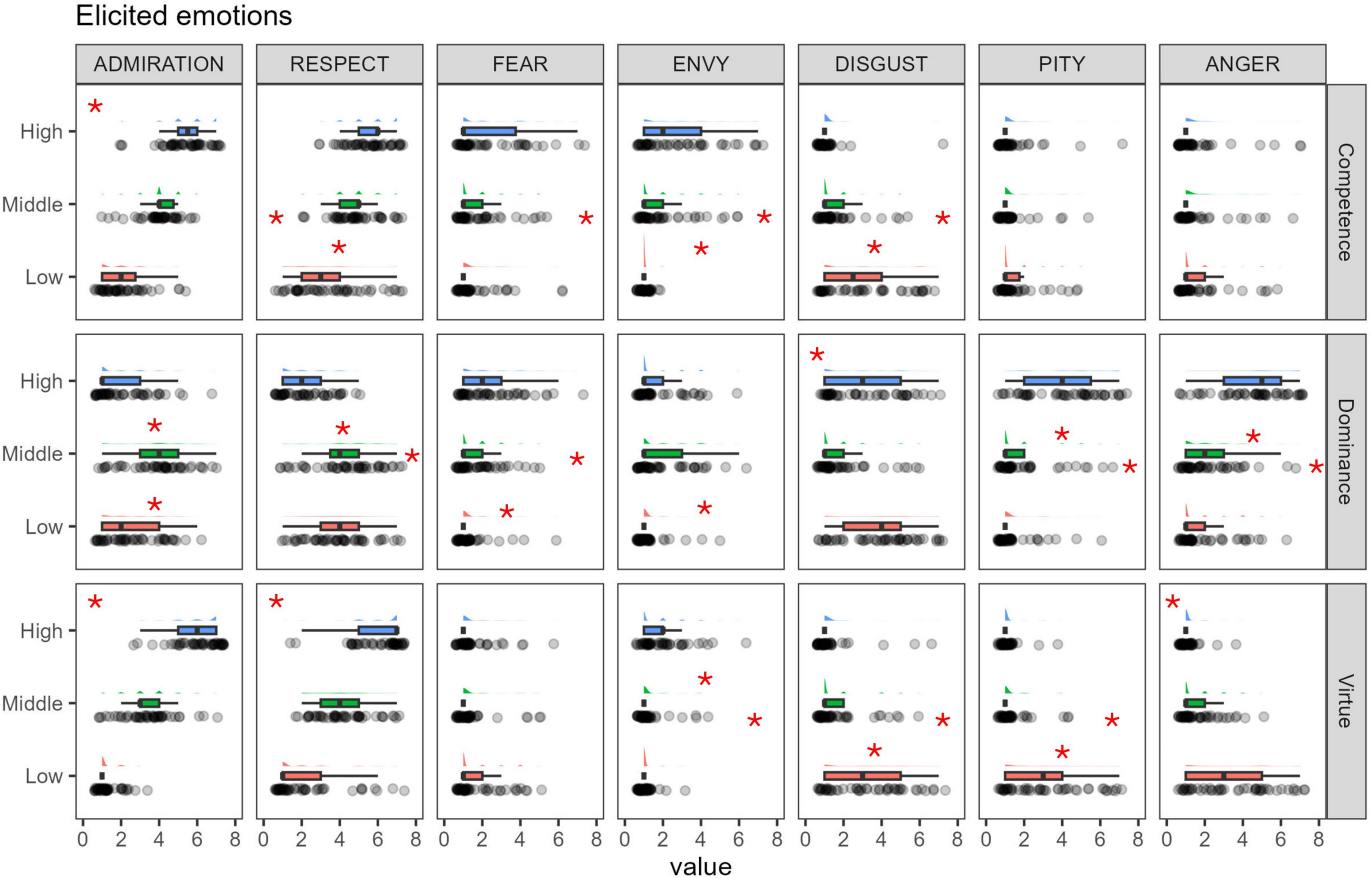
Ratings of elicited emotions for each profile and within-group comparisons. Data were analysed with ART ANOVAs [51]. Asterisks indicate significant (*p* < 0.01) post hoc comparisons. Asterisks placed in the top left corner indicate that all within-group comparisons were significant.

**Figure 6.**
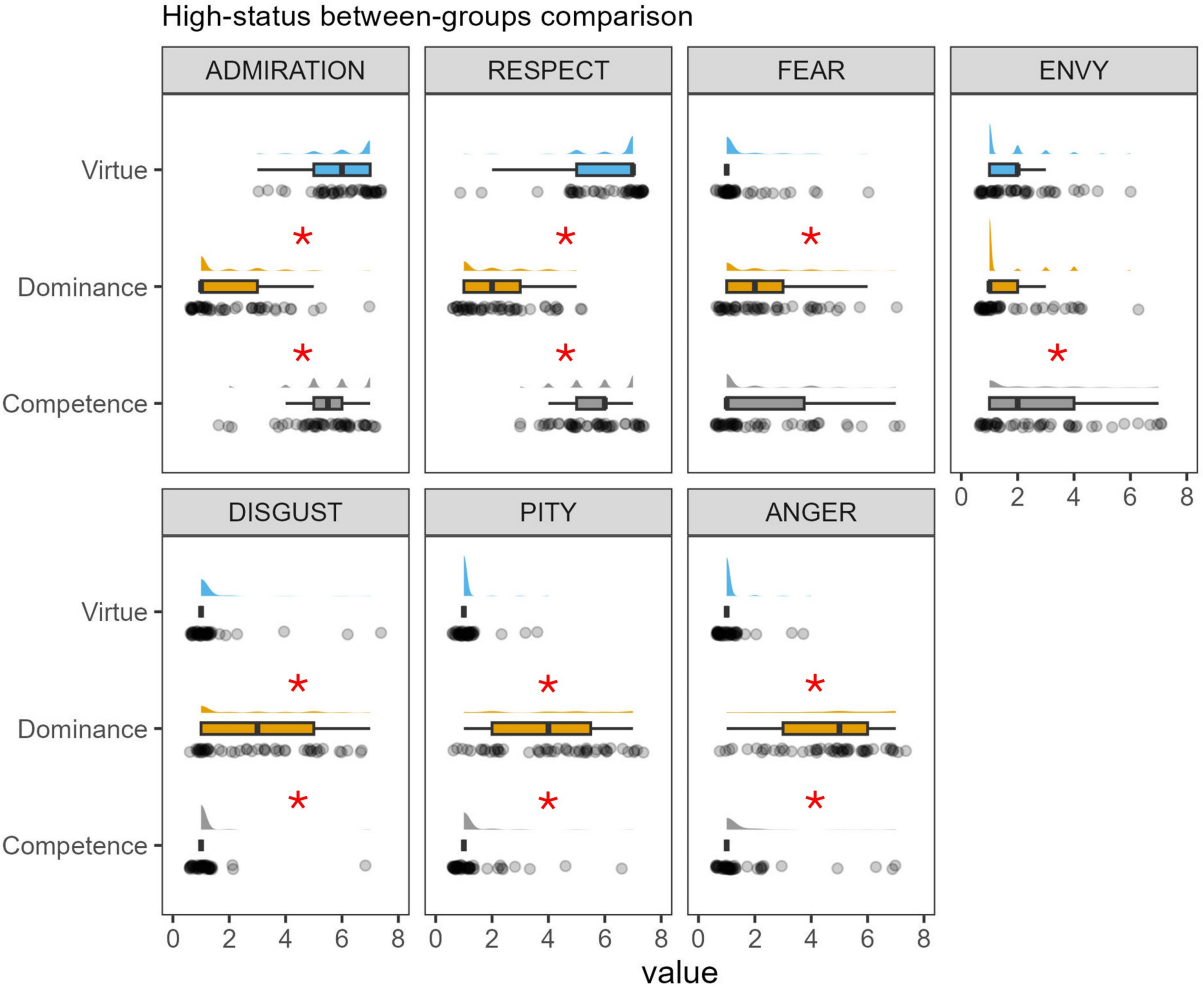
Between-group comparison of elicited emotions for the high-status profiles. Data were analysed with ART ANOVAs [51]. Asterisks indicate significant (*p* < 0.01) post hoc comparisons.

ART ANOVAs on all emotion ratings showed a significant status level × status dimension interaction (*p* < 0.0001). A full description of the within-group comparisons is provided in the electronic supplementary material, table S2. In brief, the high competent and the high virtuous profiles elicited more positive emotions (i.e. admiration and respect) compared to their middle and low-status counterparts (see also figure 5), while the high dominant and the low virtuous elicited more anger and pity compared to the other profiles in the same group. Significantly higher ratings of disgust were observed for the low competent and the low dominant profiles, while both the high competent and the high virtuous profiles elicited more envy than their counterparts. Finally, both the high competent and the high dominant elicited more fear than the other profiles in the same group. To explore how different status pathways may elicit different emotions in the observer, we looked at between-group differences for the high-status profiles (see figure 6). Admiration and respect showed a similar pattern, with significantly higher ratings for the competent compared to the dominant (admiration: estimate = 220.7, s.e. = 16.4, *t*-ratio = 13.42, *p* < 0.0001; respect: estimate = 221.68, s.e. = 19.5, *t*-ratio = 11.34, *p* < 0.0001) and lower ratings for the dominant compared to the virtuous (admiration: estimate = −253.2, s.e. = 16.5, *t*-ratio = −15.32, *p* < 0.0001; respect: estimate = −261.28, s.e. = 19.7, *t*-ratio = −13.24, *p* < 0.0001) high status. Importantly, the competent and virtuous high-status profiles elicited similar ratings of admiration (estimate = −32.5, s.e. = 16.6, *t*-ratio = −1.95, *p* = 0.57) and respect (estimate = −36.6, s.e. = 19.8, *t*-ratio = −1.94, *p* = 0.58). The high-status dominant also elicited more anger, pity and disgust compared to the competent and the virtuous (all *ps* < 0.001) and more anger compared to the virtuous (estimate = 223.5, s.e. = 19.3, *t*-ratio = 11.57, *p* < 0.01). The high competent elicited more envy than the high-status dominant (estimate = 70.52, s.e. = 18.9, *t*-ratio = 3.73, *p* < 0.01).

#### 3.2.3. Predicting lies from participants’ socioeconomic features and personality traits

**Figure 7.**
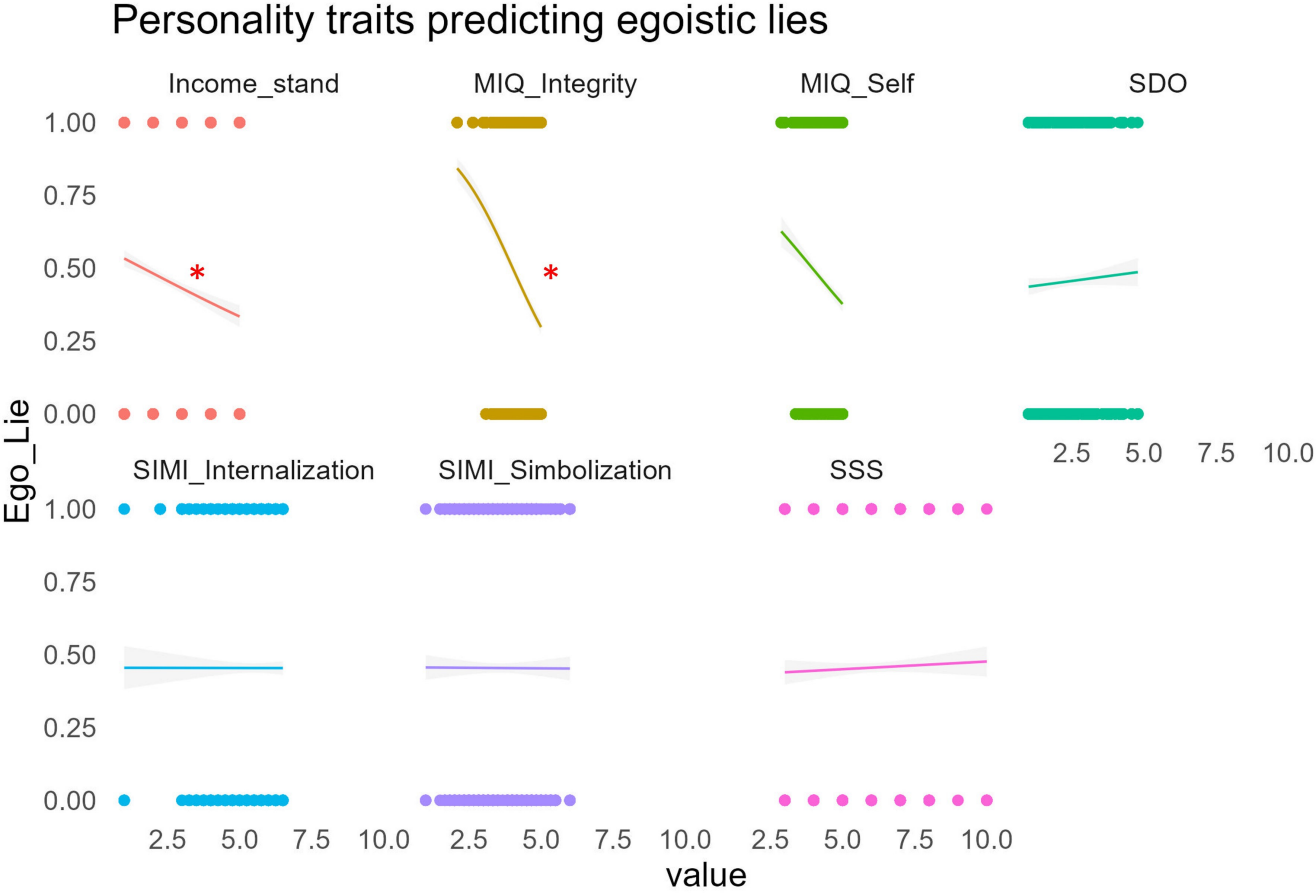
The relationship between personality traits and egoistic (i.e. self-gain) lies in the TLCG. MIQ, moral identity questionnaire; SDO, social dominance orientation; SIMI, self-importance of moral identity; SSS, subjective social status. Significant effects *p* < 0.05.

Since other-gain lies were not affected by status level or dimension, we explored the relationship between personality traits and self-gain lies through linear GLMM models including status level, status dimension and their interactions as fixed effects. The model indicated that the MIQ_integrity (*χ²* = 15.32, *p* < 0.0001) and income (*χ²* = 6.27, *p* < 0.05) negatively predicted egoistic lies, see figure 7. All the other models failed to show any significant effect.

#### 3.2.4. Predicting lies from elicited emotions

**Figure 8.**
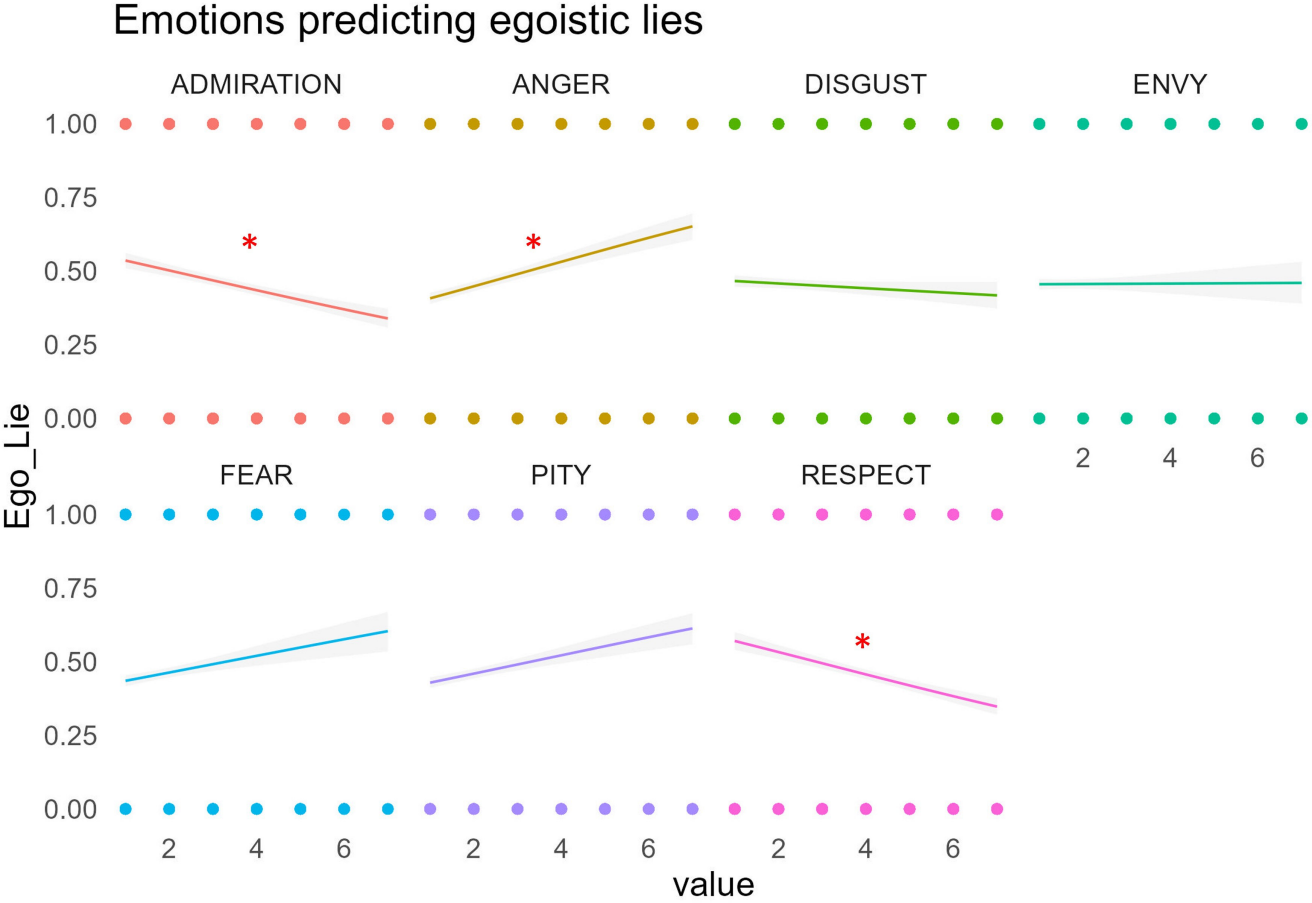
The relationship between emotions elicited by the characters (as measured in the Profile Questionnaire) and egoistic (i.e. self-gain) lies towards the same characters in the TLCG. Significant effects *p* < 0.05.

Using a similar approach, we investigated the impact of the emotions elicited by each profile on participants’ tendency to lie to that profile for self-gain. Through separate GLMMs, we observed that, after controlling for the effects of status level and status dimension, self-gain lies were positively related to ratings of evoked anger (*χ²* = 34.19, *p* < 0.0001) and negatively related to ratings of evoked admiration (*χ²* = 4.47, *p* < 0.05) and respect (*χ²* = 9.23, *p* < 0.05), see figure 8.

## 4. Discussion

This preregistered study investigated whether individuals adjust their moral behaviour based on the status of the recipient and the means through which that status was attained. Ratings of attributed status did not vary between experimental groups, supporting the notion that competence, dominance and virtue as equally effective pathways ‘to the top’ [15,40]. However, participants were less likely to lie for self-gain to opponents who attained high status using virtue compared to their low-status counterparts and compared to opponents who acquired high status through competence and dominance.

Concerning the mechanisms through which virtue may discourage deception, our findings highlight the role of positive emotions—specifically respect and admiration—which were both elevated towards the virtuous high-status individuals and negatively associated with deceptive behaviour. Virtue and prosociality are well-established determinants of status and are linked to positive emotions and favourable evaluations [36,40]. The display of moral virtue elicits other-praising emotions such as gratitude and elevation [53] and virtuous leadership is positively associated with followers’ happiness, life satisfaction, in-role and extra-role performance [54]. Therefore, our results align with previous research showing that positive emotions can motivate individuals to ‘do the right thing’ [55].

Another potential mechanism involves social learning and reciprocity: the moral stance of virtuous high-status individuals may have served as a behavioural model, prompting participants to adopt similar ethical standards [56]. In other words, participants may have been inspired by the opponent’s moral qualities and reciprocated with honest behaviour. In both scenarios, the results suggest that leaders, managers or other influential figures can promote moral behaviour in others simply by embodying ethical conduct themselves. For example, an employee completing a travel reimbursement form may be more motivated to report expenses truthfully if their manager is known to be fair, altruistic and consistently acts with integrity. The employee might even choose to omit minor personal costs, such as small incidental expenses, in an effort to mirror the ethical standards set by their manager.

Overall, the use of virtue may represent a safer option to ascend the social hierarchy, allowing to establish a positive relationship with followers and preventing the manifestation of unfavourable behaviour.

Although the dominant high-status character did not elicit more deception than the competent one, participants were significantly more likely to lie to them compared to their middle- and low-status counterparts and to the virtuous high-status. We also confirmed the proposition that while dominance can be an effective strategy for status acquisition, it is generally perceived negatively [15,57], as it elicited more negative (i.e. anger, disgust and pity) and fewer positive emotions compared to virtue and competence. Moreover, while controlling for the effects of status level and dimension, ratings of anger predicted an increase in the likelihood of egoistic lies, while respect/admiration predicted their decrease. Thus, it could be speculated that an anger-induced motivation to punish the dominant high-status opponents may be the mechanism explaining our results, even if this causal relationship was not directly tested in the present study. Although theories [39,40] predict that dominant individuals achieve status by instilling fear, ratings of evoked fear did not predict the occurrence of self-gain lies, while anger ratings did, suggesting that the observed effects are more likely to be explained by anger than fear. The absence of fear is not surprising, considering that the interaction took place online and that the dominant character posed no danger to the participants. Future research should try to replicate our study in in-person interactions, where fear may emerge in participants intimidated by dominant behaviours.

This finding bears important implications for educational and organizational research and practice, as students and employees might be more willing to lie to teachers and managers who adopt a dominant leadership style [34]. Studies from organizational and political psychology support the notion that dominant individuals can rise to leadership roles, especially in situations of conflict [58]. Nevertheless, people would not pick them as friends [59], and they tend to lose influence over time [60]. In addition, exploitative acts performed by physically dominant individuals elicit more outrage [61], while high-status dominant actors are punished more harshly for their misconduct than their prestigious counterparts [62]. In this context, the dominance strategy for attaining status and power can be seen as a risky one. By generating negative feelings among followers, it increases the likelihood of backlash or retaliation against the dominant individual whenever an opportunity arises. For example, an employee who has endured the mistreatment of a dominant boss might choose to retaliate by stealing something on their last day of work.

Contrary to our expectations, participants in the competence group did not modulate their behaviour depending on the opponent’s status level, and there was no difference in the probability of self-gain lies between the competent and dominant high-status opponents. This finding can be explained by considering that competence-based status is afforded to individuals whose outstanding abilities either provide a direct benefit to the group [25,41,63] or can be transferred through social learning [33]. In our paradigm, the competent high-status skills were essentially irrelevant for the participants, who could neither benefit nor learn from them. Therefore, it seems that, while information regarding dominance- and virtue-related behaviours adopted by individuals in previous encounters (thus in a context that is irrelevant to the observer) weighs on moral decision-making, competence-related information does not.

It is worth noting that theories of status acquisition posit that competence leads to status through respect and virtue through admiration [40], and previous experimental findings indicate that these two pathways do not interact with each other ([40], experiment 5). Our results do not support a full orthogonality, since the competent and virtuous high-status characters were rated as eliciting equal levels of respect and admiration. Rather, our findings are in line with other studies indicating that moral targets also elicit competence–respect in observers [64] and that virtuous but incompetent employees are not afforded status by their managers ([40], experiment 4), hinting to a cross-talk between respect and admiration.

One unexpected finding was that despite being provided with a dictionary-based definition of pity (see the electronic supplementary material, table S2), participants consistently rated the dominant high status and the virtuous low status opponents as eliciting high levels of this emotion. Previous work [65] defined pity as a feeling akin to disdain and contempt that entails a sense of false superiority built upon a mixture of condescension, insecurity and distancing [66]. In this sense, our results can be interpreted as an attempt by the participants to distance themselves from the two most negative characters.

Interestingly, we also observed that when the outcome of the opponents’ choice was favourable, participants took longer to lie than to tell the truth, while no difference in RTs emerged in unfavourable trials. Given that RTs are a reliable measure of the amount of cognitive conflict experienced during moral decision-making [67], we interpreted this result as an indication that lying for the benefit of others may evoke more conflict than lying for one’s own benefit. This kind of interpretation supports the idea that self-interested behaviour can be more automatic and require less cognitive control compared to behaviours aimed at benefiting others [68].

## 5. Limitations

This study has some limitations that should be noted. First, the use of vignettes describing behaviours that took place in a previous encounter may limit the procedure to one-shot, online situations. Moreover, the perception of others’ social status may differ when their behaviour is described by vignettes compared to when it is experienced in real-life interactions. Future studies should try to replicate our findings in a face-to-face setting, where the behaviour of the opponents is directly witnessed by the participants in an online or laboratory-based interaction. We also acknowledge that, although the three pathways to status can be conceived as independent, they are not mutually exclusive [40]. Indeed, characters seeking status exclusively through dominance, virtue or competence, as described in our profiles, are unlikely to be encountered in real life, where a mixture of the three strategies, mostly depending on the context, is more probably employed. For instance, individuals seeking high-status positions within an organization may rely on competence to earn the trust of their superiors, while emphasizing virtue to gain the esteem of their peers. By contrast, those aiming for leadership roles within a political party might use virtue to secure support from members of their faction and assert dominance to undermine rival leaders from other factions. In this way, moral behaviour directed towards status-seeking individuals may be affected both by the style they prioritized in a given moment and by the style they used in previous encounters.

## 6. Conclusion

With these limitations in mind, our results highlight how specific behaviours ultimately leading to status are evaluated, and how these behaviours weigh on moral decision-making. While the moral exclusion theory [9] suggests that individuals with high instrumental value are more likely to be treated fairly, our findings indicate that status alone may not be enough to prevent exclusion from the ‘scope of justice’. Instead, the pathway through which status is attained appears to play a more crucial role. Together with previous findings [13,21], this result underscores the importance of the recipient’s social identity in shaping moral decision-making—a factor that, as Hester & Gray [69] point out, is often neglected in moral psychology’s pursuit of universal principles of ethical behaviour. While this issue has been explored in the context of moral judgement (see [70]), our study extends it to first-person moral behaviour, highlighting the critical influence of the recipient’s status level and the means by which that status was attained on the likelihood of self-serving lies.

## Data Availability

The hypotheses and methods were preregistered (https://osf.io/xjkfp/view_only=42dffbed4bb94e8b98a047e7379d8539) on 3 December 2021, prior to data collection which began on 15 December 2021. The analysis plan was preregistered. There were no deviations from the preregistration. All study materials, data and codes are publicly available https://osf.io/e5wz7/?view_only=72e96a6ccbc64cdc89cd8918a1874f1c . Supplementary material is available online [71].
